# Population Pharmacokinetics of Tafenoquine, a Novel Antimalarial

**DOI:** 10.1128/AAC.00711-18

**Published:** 2018-10-24

**Authors:** Nilay Thakkar, Justin A. Green, Gavin C. K. W. Koh, Stephan Duparc, David Tenero, Navin Goyal

**Affiliations:** a Clinical Pharmacology Modeling and Simulation, GlaxoSmithKline Research and Development, King of Prussia, Pennsylvania, USA; b GlaxoSmithKline Global Health Group, Stockley Park, Uxbridge, United Kingdom; c Medicines for Malaria Venture, Geneva, Switzerland

**Keywords:** tafenoquine, antimalarial, 8-aminoquinoline, population pharmacokinetics

## Abstract

Tafenoquine is a novel 8-aminoquinoline antimalarial drug recently approved by the U.S. Food and Drug Administration (FDA) for the radical cure of acute Plasmodium vivax malaria, which is the first new treatment in almost 60 years. A population pharmacokinetic (POP PK) analysis was conducted with tafenoquine exposure data obtained following oral administration from 6 clinical studies in phase 1 through phase 3 with a nonlinear mixed effects modeling approach. The impacts of patient demographics, baseline characteristics, and extrinsic factors, such as formulation, were evaluated. Model performance was assessed using techniques such as bootstrapping, visual predictive checks, and external data validation from a phase 3 study not used in model fitting and parameter estimation. Based on the analysis, the systemic pharmacokinetics of tafenoquine were adequately described using a two-compartment model. The final POP PK model included body weight (allometric scaling) on apparent oral and intercompartmental clearance (CL/*F* and *Q*/*F*, respectively), apparent volume of distribution for central and peripheral compartments (*V*_2_/*F* and *V*_3_/*F*, respectively), formulation on systemic bioavailability (*F*1) and absorption rate constant (*K_a_*), and health status on apparent volume of distribution. The key tafenoquine population parameter estimates were 2.96 liters/h for CL/*F* and 915 liters for *V*_2_/*F* in P. vivax-infected subjects. Additionally, the analyses demonstrated no clinically relevant difference in relative bioavailability across the capsule and tablet formulations administered in these clinical studies. In conclusion, a POP PK model for tafenoquine was developed. Clinical trial simulations based on this model supported bridging the exposures across two different formulations. This POP PK model can be applied to aid and perform clinical trial simulations in other scenarios and populations, such as pediatric populations.

## Abstract (precis)

Tafenoquine is a novel 8-aminoquinoline antimalarial drug recently approved by the U.S. Food and Drug Administration (FDA) for the radical cure of acute Plasmodium vivax malaria, which is the first new treatment in almost 60 years.

## INTRODUCTION

There is a significant global disease burden of malaria due to Plasmodium vivax. In 2015 alone, the World Health Organization (WHO) reported an estimated 8.5 million (uncertainty range, 6.6 million to 10.8 million) new cases of P. vivax infection (1). The current standard for the treatment of P. vivax malaria includes chloroquine administration for 3 days followed by 15 mg primaquine once daily for 14 days to prevent relapse; this dose may be increased up to 30 mg once daily primaquine for 14 days in some cases (2, 3). This 14-day regimen of primaquine leads to poor patient compliance (4). Additionally, primaquine can cause hemolytic toxicity in subjects with glucose-6-phosphate dehydrogenase (G6PD) deficiency (5). This poor patient compliance leads to significant rates of relapse, highlighting the need for an alternative antirelapse treatment.

Tafenoquine (TQ; SB-252263 or WR238605) is a novel 8-aminoquinoline antimalarial drug being developed for the radical cure of acute P. vivax malaria by coadministration as a single dose with standard doses of chloroquine (CQ). Importantly, tafenoquine possesses activity against all stages of the Plasmodium life cycle, including the hypnozoites of P. vivax. Tafenoquine has a long elimination half-life (approximately 15 days).

To date, multiple clinical studies have been conducted to evaluate the safety, exposure, and efficacy of tafenoquine. The studies range from phase 1 healthy volunteer studies with dense pharmacokinetic (PK) sampling to phase 3 patient studies with a sparse PK sampling scheme. It is important to understand the impact of subject demographics and other relevant clinical variables that may affect tafenoquine systemic exposure and thus influence the drug dosing paradigm. In addition, as the investigation of tafenoquine progressed, the phase 3 studies employed a tablet formulation, whereas the earlier studies, including the phase 2B dose-ranging study, utilized a capsule formulation (6).

In these analyses, we aimed to characterize the population pharmacokinetics (POP PK) of tafenoquine administered orally, in addition to the influence of subject demographics and disease status and the impact of the formulations used. The analyses utilized the most recent clinical studies that would provide data obtained after sampling over the earlier and later time points to help adequately characterize the exposures (e.g., maximal plasma concentration [*C*_max_] and area under the concentration-time curve [AUC] from 0 h to time *t* [AUC_0–*t*_]). The population PK approach allows combining data obtained across different doses, sampling strategies, subject demographics, health statuses, formulations, and other variables to adequately characterize tafenoquine population PK (POP PK) (7).

## RESULTS

### Population demographics.

A total of 5,286 tafenoquine systemic observations from 675 subjects receiving tafenoquine were included in the analysis (parameter estimation) data set. These data were collected from 5 studies (a drug-drug interaction [DDI] study, a stable isotope label [SIL] study, a thorough QTc [TQT] study, and the DETECTIVE part 1 and DETECTIVE part 2 studies), summarized in Table 1. The PK data from the GATHER study were not utilized in the estimation data set but were kept separate to validate the final POP PK model performance. A summary of subject demographics and key clinical covariates is provided in Table 2. Briefly, 28.6% of subjects were healthy volunteers and 71.4% were patients. Forty-four percent of subjects were administered a tablet formulation of tafenoquine, while the remaining 56.0% of subjects received a capsule formulation of tafenoquine. A summary of the demographics for the GATHER (patient study) data is provided in Table S1 in the supplemental material. These demographics are highly comparable to the DETECTIVE part 2 (patient study) demographics.

**TABLE 1:** Summary of data used for population PK analysis and external model validation^*a*^

Study	Study phase	No. of subjects	No. of PK samples	Tafenoquine dose(s) studied (mg)	Formulation	Population by health status
200951 (DDI)	1	24	283	300	Tablet	Healthy volunteers
201780 (SIL)	1	14	232	300	Tablet	Healthy volunteers
TAF114582 (TQT)	1	155	2,215	300, 600, 1,200	Capsule	Healthy volunteers
TAF112582 (DETECTIVE part 1)	2B	223	1,067	50, 100, 300, 600	Capsule	Patients
TAF112582 (DETECTIVE part 2)	3	259	1,489	300	Tablet	Patients
TAF116564 (GATHER)	3	166	1,001	300	Tablet	Patients

a No. of subjects, the number of subjects from the tafenoquine-only arms that were included in the current analyses; DDI, drug-drug interaction study; SIL, stable isotope label study; TQT, thorough QTc study. The detailed sampling scheme is listed in Table S3 in the supplemental material.

**TABLE 2:** Summary of demographics for subjects included in the analysis data set^*a*^

Characteristic	Median (range)	Category	No. (%) of subjects
Age (yr)	35.0 (15.0–79.0)		
Wt (kg)	69.3 (37.2–138)		
Body mass index (kg/m^2^)	24.3 (15.5–47.0)		
Gender		Female	171 (25.3)
		Male	504 (74.7)
Health status		Healthy volunteers	193 (28.6)
		Patients	482 (71.4)
Formulation status		Tablet	297 (44.0)
		Capsule	378 (56.0)
Race		Caucasian	95 (14.1)
		African American	123 (18.2)
		Asian	160 (23.7)
		American Indian/Alaska native	193 (28.6)
		Other	1.00 (0.10)
		Multiple	103 (15.3)

a This table includes data from the 5 studies (the DETECTIVE part 1, DETECTIVE part 2, drug-drug interaction [DDI], stable isotope label [SIL], and thorough QTc [TQT] studies).

### Population PK model development.

A two-compartment model with first-order absorption and elimination was used as the starting POP PK model based on a better fit, the observed biphasic profile, and previous analysis (8). Allometric weight-based scaling was applied to all clearance and volume PK parameters (apparent oral clearance [CL/*F*], the apparent volume of distribution for the central compartment [*V*_2_/*F*], intercompartmental clearance [*Q*/*F*], and the apparent volume of distribution for the peripheral compartment [*V*_3_/*F*]). These parameters were also in agreement with the previously published results from a study with a smaller sample size (8). A one-compartment model has been previously reported for tafenoquine (9, 10). However, the dosing regimen and PK sampling in those studies may not have allowed for an adequate characterization of tafenoquine's biphasic profile observed with these single-dose data, which included dense early-phase PK sampling from some studies. This two-compartment model with allometric scaling was considered the base model for all further analyses. Following base model development, further evaluations were undertaken to examine the impact of covariates and other differences in the populations between the different studies included in the analysis data set.

### Covariate analysis.

The selection of key covariates in model evaluation was based on physiological relevance, understanding the key differences across populations and studies. Notable differences across studies were the formulations used (tablets versus capsules) and health status (healthy volunteers versus patients). Consequently, exploratory covariate-versus-ETA (between-subject variance) plots were generated to evaluate any bias across these and other covariates. Drug formulation was introduced as a covariate on bioavailability (*F*1), with tablet being used as the reference formulation (i.e., *F*1 for the tablet formulation [*F*1_tablet_] = 1) along with formulation-specific absorption rate constants. This led to a 122.5-point drop in the objective function value (OBJFV). There was no improvement in model fit or OBJFV with the inclusion of formulation-specific ETAs (the between-subject variability parameter). The difference in bioavailability between these two formulations based on this model was less than 20%, with the point estimate for capsule relative bioavailability being 0.833 (i.e., *F*1 for the capsule formulation [*F*1_capsule_] = 0.833). Addition of drug formulation on the absorption rate constant (*K_a_*) led to a 7.364-point drop in OBJFV.

A key covariate across these studies was the health status of the subjects. The DDI, SIL, and TQT studies were healthy volunteer studies, while the DETECTIVE (parts 1 and 2) and GATHER studies were patient studies. The ETA(*V*_2_/*F*)-versus-health status plot stratified by the formulation demonstrated bias (Fig. S1). The visual predictive check (VPC) of the DDI study also demonstrated overprediction of exposures at the early time points. Addition of health status as a covariate on *V*_2_/*F* and *V*_3_/*F* led to a drop in OBJFV of 109.2 and 231.3 points, respectively, along with an improvement in the diagnostic ETA(*V*_2_/*F*) plot (Fig. S1). The VPC plots also demonstrated an improved prediction for the healthy subjects (Fig. S1). On the basis of the collective criteria described above, other covariates, such as age and ethnicity, were also evaluated and did not demonstrate any relevant impact on tafenoquine PK.

Backward elimination was then performed to test each covariate in the full model. All covariates except the effect of formulation on *K_a_* led to a significant increase in OBJFV upon their removal and were retained in the model. While the OBJFV improved by eliminating the impact of formulation on *K_a_* (20.4 points), there were negligible changes in the POP PK parameter estimates. However, while the *K_a_*s for the two formulations were highly comparable, use of the formulation-specific *K_a_* allowed for a better approximation of the formulation-specific *C*_max_. One of the objectives of this POP-PK analysis was to compare the two formulations with respect to their *C*_max_ and AUC. Thus, the formulation-specific *K_a_* was retained in the final model. Nevertheless, as can be seen from the values listed in Table 3, the formulation-specific absorption rate constants were highly comparable across the two formulations.

**TABLE 3:** Population PK parameters for the final model and bootstrap results^*a*^

Parameter	Value(s) for final model parameters		
	Final run	Bootstrap (*n* = 500 runs)	
		Median estimate	90% CI
CL/*F* (liters/h)	2.96	2.96	2.87–3.05
*V*_2_/*F* (liters)	915	913	879–956
*Q*/*F* (liters/h)	5.09	5.10	4.76–5.43
*V*_3_/*F* (liters)	664	665	634–692
Absorption lag time (h)	0.908	0.930	0.904–0.950
*K_a_* (h^−1^)	0.252	0.254	0.226–0.296
Capsule effect on *K_a_*	0.924	0.914	0.805–1.03
Relative bioavailability (capsule)	0.863	0.866	0.833–0.900
*V*_2_/*F* ratio (healthy volunteers/patients)	1.35	1.35	1.30–1.41
*V*_3_/*F* ratio (healthy volunteers/patients)	0.347	0.340	0.295–0.396
IIV CL/*F*	32.1	32.0	30.0–34.1
IIV *V*_2_/*F*	34.4	34.3	31.8–37.1
IIV CL-*V*_2_ block	33.3	29.9	27.7–32.5
IIV *K_a_*	40.4	39.6	31.7–48.0
IIV ALAG1	44.3	43.4	38.8–56.1
IIV error	33.0	33.2	27.2–38.5
Random residual variability (% CV)	15.0	14.9	14.3–15.8

a *K_a_*, absorption rate constant; CL/*F*, oral clearance from the central compartment; *V*_2_/*F*, volume of distribution for the central compartment; *V*_3_/*F*, volume of distribution for the peripheral compartment; *Q*/*F*, intercompartmental clearance; IIV, interindividual variability, expressed as the percent coefficient of variation; CI, confidence interval. The tablet formulation was considered the reference, i.e., *F*1_tablet_ = 1.

### Final model.

**FIG 1:**
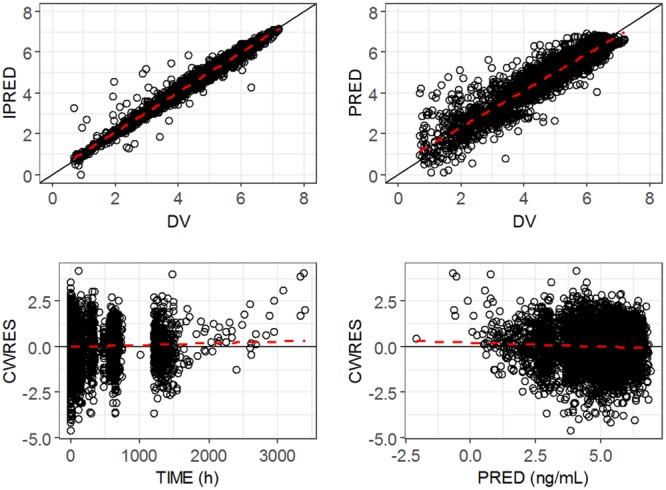
Goodness-of-fit plots for the final model. The circles represent the observed data (DV), individual predictions (IPRED), population predictions (PRED), and conditional weighted residuals (CWRES). The solid line represents the line of unity, and the dashed red line represents the trend line for the corresponding data.

The final model was a two-compartment model with allometrically scaled weight as a covariate on CL/*F*, *V*_2_/*F*, *Q*/*F*, and *V*_3_/*F*; formulation status as a covariate on *F*1 and *K_a_*; and health status as a covariate on *V*_2_/*F* and *V*_3_/*F*. The parameter estimates along with the bootstrap results from the final POP PK model are summarized in Table 3. The covariance step was aborted due to rounding errors, and the parameter precision was characterized by the bootstrap estimates. The ETA shrinkages for key variables, *viz*., CL, *V*_2_, *K_a_*, ERR (error), and ALAG1 (absorption lag time), were 3.9, 1.6, 18.2, 3.2, and 54.3%, respectively, while the ETA shrinkage for residual variability was 8.1%. While there are no established cutoff values for shrinkage, lower estimates indicate adequate data to estimate the corresponding intersubject variability estimates reliably. The high shrinkage value for ALAG1 compared to that obtained from the healthy volunteers could thus be potentially related to a lack of dense early PK sampling in patient studies. The goodness-of-fit plots from this final model are shown in Fig. 1. The model diagnostics including the residual plots demonstrate an adequate fit to the observed data at the population and individual levels.

### Population PK model performance.

The final model was qualified by several approaches, such as bootstrapping and VPCs. This was conducted to ensure the internal stability of the model and the ability to explain the observed data and the predictive power for a study whose data were not included in model building (GATHER study).

### Bootstrapping.

Five hundred data sets were generated by resampling subject-level data from the analysis data set. The parameter estimates were generated for the final model against each of these 500 data sets. The median and 90% confidence intervals of the parameter estimates from the bootstrap run were compared with the estimates from a single model run and are listed in Table 3. The bootstrap results demonstrate adequate stability of the parameter estimates.

### Visual predictive checks.

The VPC of the final model was performed using parameter estimates from the 500 bootstrap runs. Each of the bootstrap run estimates was used for simulating a clinical trial using the mrgsolve package in R. Thus, 500 clinical trials were simulated and summarized across studies, doses, and formulations. The simulation data set contained the same subject demographics employed in the estimation data set. A dense sampling grid was utilized to generate the VPC plots.

**FIG 2:**
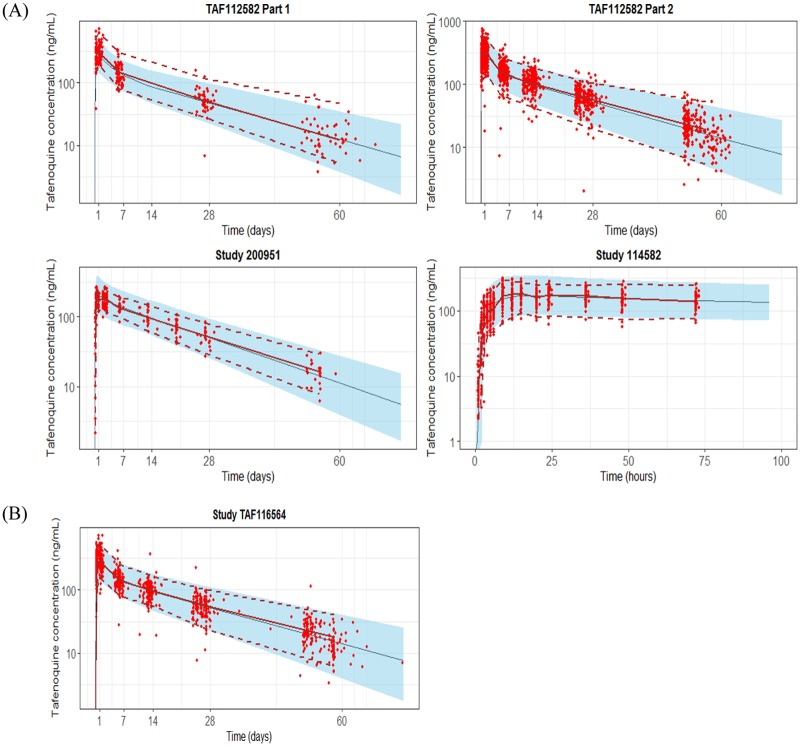
Visual predictive checks for the final model across different studies and formulations at the 300-mg dose (A) and for the TAF116564 (GATHER) study for external model validation (B). The blue bands and lines represent the 95% prediction intervals and median predictions, respectively. The red dots and red lines represent the observed data and observed medians, respectively.

Simulating clinical trials with individual bootstrap vectors enabled simulation with parameter uncertainty. The VPCs for some studies at the 300-mg dose are presented in Fig. 2A, which demonstrated that the model predictions adequately captured the observed concentration-time points and trends across different doses, studies, and populations (healthy volunteers and patients) within the 2.5th and 97.5th percentiles of the simulated values. The model fit for all other doses and studies are presented in Fig. S2.

### External validation.

The GATHER study (phase 3) data were not utilized in the model building and thus were utilized as a data set for external validation of the final POP PK model. The final model was utilized to simulate tafenoquine concentrations in a study population whose demographics reflected those of the population in the GATHER study. Five hundred clinical trials were simulated using bootstrap parameter estimates with a simulation data set comprising data for the GATHER study subjects and a dense sampling grid. A VPC plot from the simulation is displayed in Fig. 2B, demonstrating that the model was able to predict the GATHER study systemic exposure well. The external data set validation provided further confidence in the predictive power of the final POP PK model.

### Relative bioavailability across formulations.

The POP PK model was employed to bridge the exposures of the capsule and tablet formulations administered across different studies. The relative bioavailability across tablet and capsule formulations was assessed using different approaches and provided a comparison of the systemic tafenoquine exposures across the DETECTIVE part 1 (capsule formulation), DETECTIVE part 2 (tablet formulation), and GATHER (tablet formulation) studies. The results obtained from the three approaches to compare the relative bioavailability across formulations are summarized below.

### Relative bioavailability for bootstrapped final model.

Based on the final model, the relative bioavailability point estimate of capsules compared to that of tablets was estimated to be 86% (*F*1_capsules_ = 0.86). The 90% interval on the relative bioavailability estimate for capsules (*F*1_capsules_) from the 500 bootstrap runs was 0.83 to 0.90, well within the traditional bioequivalence (BE) limits of 0.8 to 1.25.

### Exposures obtained from *post hoc* estimates.

**FIG 3:**
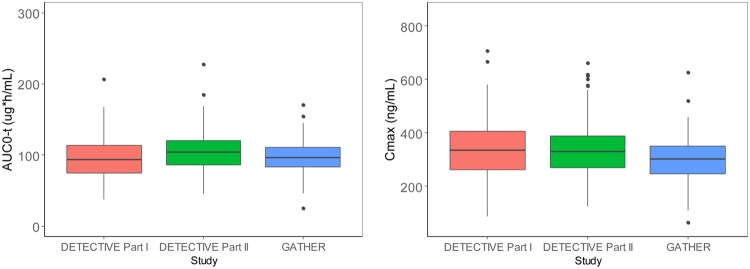
Comparisons of *post hoc* exposure estimates (AUC_0–60_ and *C*_max_) across studies at the 300-mg dose. The lower and upper hinges of the box plot correspond to the first and third quartiles, respectively (25th and 75th percentiles, respectively), and the line represents the median. The upper and the lower whiskers represent the 95% confidence intervals. AUC_0–*t*_ summarized the AUC up to day 60.

The final POP PK model was used to obtain *post hoc* individual PK parameters (CL, *V*_2_, *Q*, *V*_3_, *F*1, and ALAG1). A new population with demographics similar to those of the subjects in the analysis and validation data sets and intense sampling time points was created and merged with the individual *post hoc* PK parameter estimates for those subjects. The predicted concentrations were used to compute the AUC from 0 to 60 days (AUC_0–60_) and *C*_max_ values for the clinically recommended 300-mg dose. The results demonstrated that the exposures between the studies using different formulations were highly comparable (Fig. 3). This statistical analysis utilized the log-transformed AUC and *C*_max_ parameters and was similar to the analysis that is usually performed in a relative bioavailability/bioequivalence study per regulatory guidance (11). The results of the analysis analogous to that performed in a traditional bioequivalence study outcome are provided in Table 4. The point estimates and 90% confidence intervals of the ratios of the tablet versus capsule AUC_0–60_ and *C*_max_ are entirely within the traditional bioequivalence limits of 80 to 125%.

**TABLE 4:** Statistical analysis of *post hoc* estimates^*a*^

Comparison	Point estimate (90% CI) of the ratio	
	AUC_0–60_	*C*_max_
DETECTIVE part 2 (tablet) vs DETECTIVE part 1 (capsule)	1.07 (1.00–1.14)	1.00 (0.93–1.08)
GATHER (tablet) vs DETECTIVE part 1 (capsule)	1.00 (0.94–1.07)	0.90 (0.83–0.98)

a AUC_0–60_, the AUC from 0 to 60 days; CI, confidence interval.

### Predicted exposures obtained from 500 bootstrap model runs.

Another approach involved simulating from parameter uncertainty. Systemic tafenoquine concentrations were predicted for different formulations across studies using population estimate vectors generated from the 500 bootstrap runs. The AUC_0–60_ and *C*_max_ values assessed for each subject across each trial and each replicate are summarized and listed in Table S2. The results demonstrate comparable exposures across the capsule and tablet formulations utilized in tafenoquine clinical trials.

All the above-described approaches demonstrated a lack of any clinically relevant difference in the systemic exposures across the capsule and tablet formulations used in these clinical studies.

## DISCUSSION

Tafenoquine is the first new treatment approved in more than half a century for the radical cure of P. vivax malaria. The POP PK of tafenoquine were characterized from systemic tafenoquine exposure after oral administration. The most recent clinical studies were selected to provide adequate data to help characterize the exposures from studies utilizing different populations, doses, formulations, and PK sampling schemes. The population analysis approach using nonlinear mixed effects modeling allowed pooling of the data from different studies with various sampling and dosing schemes to reliably estimate key PK parameters and associated variability estimates. The POP PK model was developed to understand the impact of subject demographics, formulation, and other relevant variables on tafenoquine PK.

The results of the tafenoquine POP PK analysis showed that a two-compartment model adequately described the PK data. The final POP PK model included body weight (allometric scaling) on CL/*F*, *V*_2_/*F*, *Q*/*F*, and *V*_3_/*F*; formulation on *F*1 and *K_a_*; and health status on *V*_2_/*F* and *V*_3_/*F* (12, 13). Body weight is known to be correlated with clearance processes, and thus, body weight is a common predictor of CL/*F* for many drugs. The final model with allometric scaling on all clearance and volume of distribution parameters will be helpful for the broader application of this model across other populations (e.g., for characterizing pediatric exposure) (ClinicalTrials.gov identifier NCT02563496).

Formulation differences can potentially result in significant exposure differences. The differences may affect drug absorption, impacting the systemic *C*_max_ and/or AUC values. Population analyses can be effectively utilized to characterize any such differences with appropriate data. The current analyses identified that while formulation impacted the relative bioavailability, the tafenoquine exposures were highly comparable across the tablet and capsule formulations utilized in clinical studies.

Health status was included as a covariate on the apparent volume of distribution. The difference in the volume of distribution between the healthy volunteers and patients could be attributed to different reasons. A previous analysis in healthy volunteers demonstrated that subjects receiving chloroquine as background medication (as in the patient studies DETECTIVE part 1 and part 2 and GATHER) had a higher tafenoquine *C*_max_ than subjects administered tafenoquine alone (14). The base model (which did not include health status as a covariate) can be considered to assume the chloroquine background in all study subjects, including in healthy volunteers, which may in part explain the overprediction of tafenoquine concentrations at earlier time points in these healthy volunteers. A lower volume of distribution corresponds to higher concentrations, and the volume of distribution in healthy volunteers in the analyses data set may need to be corrected for this. Accordingly, when evaluated for adjustment in the volume of distribution, the model demonstrated that healthy volunteers had an approximately 30% higher *V*_2_ [*V*_2_ for healthy subjects = 1.3] than the malaria patients. Thus, the higher concentrations in patients than in healthy volunteers may be due to the chloroquine background therapy.

Another plausible explanation is that the patients with malaria infection may be suffering from various levels of dehydration, thus having a lower volume of distribution than healthy volunteers. In other words, healthy volunteers are appropriately hydrated and thus have a higher volume of distribution. This is consistent with the findings of a previous study which showed that renal chemistry markers are often altered in malaria patients, suggesting significant dehydration (6). Health status was also found to be a significant covariate on the peripheral volume of distribution, which may likely be due to the differences in the volume of distribution in the peripheral compartment (*V*_3_), based on whether the subject is healthy or experiencing malaria infection. A possibility may be that malaria infection upsets the basement membrane or cell-cell interactions, resulting in a leakier environment, reflected as a higher *V*_3_ in the patient population.

The model-building exercise demonstrated a lack of an effect of demographics, such as age, gender, and ethnicity, on tafenoquine PK. Based on the wide range of demographic characteristics of the subjects included in the analysis, the tafenoquine PK behavior is expected to be similar across subjects of all ethnicities, ages, and genders. The lack of a clinically significant difference in exposures across different ethnicities has also been reported for other antimalarial compounds (15). The model performance was evaluated by various methods, and all approaches supported adequate model qualification. The predictive power of the model was established by external validation of the tafenoquine systemic PK data from the phase 3 GATHER study, which were not used in the analyses/estimation step. This demonstrated the model utility in reliably predicting the systemic tafenoquine exposures across existing and future studies.

Importantly, the POP PK model was successfully applied to characterize relative bioavailability across formulations to assess any exposure differences between capsules administered to patients in the phase 2B dose-ranging DETECTIVE part 1 study and tablets administered to patients in the phase 3 studies DETECTIVE part 2 and GATHER. The POP PK analyses results demonstrated that the relative bioavailability of tablets and capsules was highly comparable and within the traditional BE limits of 80 to 125%. The exposure (*C*_max_ and AUC) comparisons with the two formulations across studies using various approaches all corroborated a lack of a clinically relevant difference in bioavailability across the capsule and tablet formulations.

The clinical trial simulations based on the POP PK model can be used to answer multiple other questions. The analysis briefly described bridging PK across formulations, demonstrating its utility as a powerful tool in drug development, obviating the need for a formal BE study and thereby avoiding unnecessary human drug exposure and conserving the ever-shrinking resources in drug research and development. The model has also been applied to design clinical trials in special populations, such as the ongoing tafenoquine pediatric study (ClinicalTrials.gov identifier NCT02563496).

In summary, a population PK model for tafenoquine was successfully developed and qualified using data from phase 1 through phase 3 clinical studies. No dose adjustment is warranted in tafenoquine dosing based on demographic characteristics, such as age, gender, or ethnicity. One additional application of this model was to bridge exposures across different formulations, negating the need for a formal clinical study. The results based on the final POP PK model demonstrated that there are no clinically relevant differences in the relative bioavailability between the tablet and capsule formulations. This population PK model can also be applied to aid and perform further clinical trial simulations in other scenarios and populations, such as pediatric populations.

## MATERIALS AND METHODS

### Study design and population.

The data used in the POP PK analysis and validation were obtained from a total of six randomized, parallel-design studies. Tafenoquine was administered as a single dose in all studies, with the exception of the thorough QTc (TQT) study (16). In the TQT study, tafenoquine was administered as a single dose for the 300- and 600-mg cohorts. For the 1,200-mg supratherapeutic dose, tafenoquine was administered as 400 mg once daily for 3 days.

All patients and/or their legally authorized representatives were required to give written, informed consent. The studies were approved by local review boards/ethics committees and were conducted in accordance with the Declaration of Helsinki and Good Clinical Practice guidelines.

Data from five studies TAF112582 part 1 and part 2 [henceforward referred to as DETECTIVE part 1 and DETECTIVE part 2, respectively; ClinicalTrials.gov identifier NCT01376167]; study 200951 henceforward referred to as the drug-drug interaction [DDI] study [ClinicalTrials.gov identifier NCT02184637]; study 201780 (henceforward referred to as the stable isotope label [SIL] study [ClinicalTrials.gov identifier NCT02751294]; study TAF114582 henceforward referred to as the TQT study [ClinicalTrials.gov Identifier NCT01928914]) were included for the POP PK analyses (6, 17). Data from the TAF116564 study (henceforward referred to as the GATHER study; ClinicalTrials.gov identifier NCT02216123) were used for the external model validation. A brief summary of key variables across these studies is summarized in Table 1.

### Bioanalysis.

Human plasma samples were analyzed for tafenoquine using a validated analytical method based on protein precipitation, followed by high-performance liquid chromatography-tandem mass spectrometry (LC-MS-MS) analysis. For all studies except the SIL study, the lower limit of quantification (LLQ) for tafenoquine was 2 ng/ml and the higher limit of quantification (HLQ) was 3,000 ng/ml. Quality control samples (QC), prepared at 4 different analyte concentrations and stored with the study samples, were analyzed with each batch of samples against separately prepared calibration standards. For the analysis to be acceptable, no more than one-third of the QC results could deviate from the nominal concentration by more than 15%, and at least 50% of the results from each QC concentration should be within 15% of nominal. The applicable analytical runs met all predefined run acceptance criteria.

For the SIL study, human plasma samples were analyzed for tafenoquine and tafenoquine M+5 (SIL) concentrations using a validated analytical method based on protein precipitation, followed by LC-MS-MS analysis. The LLQ for tafenoquine and tafenoquine M+5 was 0.5 ng/ml using a 7-μl aliquot of human plasma, and the HLQ was 500 ng/ml.

### Population PK model development.

The POP PK analysis was performed using NONMEM software, version 7.3.0 (ICON Development Solutions), and run management was performed using Pirana software (version 2.9.0) on an in-house model-based analysis platform (MAP; Rudraya Corporation) (18). The POP PK analysis was based on the principles highlighted in the U.S. Food and Drug Administration (7) and European Medicines Agency (19) population pharmacokinetic regulatory guidance. Bootstrapping was performed using Perl-speaks-NONMEM (version 3.7.6) (20). Visual predictive checks were performed using mrgsolve (21). All data preparation, summary statistics, and data visualization were performed using validated SAS software (version 9.2 or higher) and/or R (version 3.3.2) with RStudio (version 0.99.902) with ggplot2 (22, 23). Less than 4% of the nondosing observed concentrations were below the quantification limit (BQL) or nonquantifiable (NQ). Given the small amount of BQL data, the BQL observations were excluded from the analysis.

For the POP PK analysis, a two-compartment structural model was utilized as a starting point, based on the previous PK analysis of the DETECTIVE part 1 data (8). Log concentrations were utilized for model building to provide model stability in this analysis with tafenoquine concentrations ranging from 2.13 to 1,013 ng/ml. The interindividual variability (IIV) parameter was evaluated for other population parameters (e.g., *Q*/*F*, *V*_3_/*F*) without any significant improvement in model fit or a drop in the objective function value (OBJFV). Thus, it was not included in the model and further correlations between IIV terms were not assessed. An additive residual error with IIV was used to describe the residual variability. The additive error with the log-transformed data reflected an exponential residual error model. The GATHER study data set was not utilized for PK parameter estimation with the analysis data set but was used for external validation to evaluate the model performance of the final POP PK model.

### Covariate analysis.

Covariates were evaluated in this POP PK analysis to assess the impact of key demographics, the formulation, and other relevant variables on drug exposure in subjects. Covariate analysis was primarily driven by collective evaluation of physiological relevance, goodness-of-fit plots, a drop in the objective function value (OBJFV), visual predictive checks (VPCs), parameter estimates, variability, and their precision. The following covariates were evaluated in the POP PK model: body weight, age, ethnicity, gender, formulation, and health status.

The relationship between body weight and the PK parameters was evaluated using an allometric relationship. Fixed exponents of 0.75 and 1 were applied for the clearance and volume parameters, respectively (12, 13). Other continuous and categorical covariates were tested using a power model centered using the median covariate value for that sample, as shown in equations 1 and 2.

(1) $$P_{ij}=\theta_{\text{pop},j}\cdot (\frac{{\mathrm{cov}}_{\text{ind}}}{{\mathrm{cov}}_{\text{med}}}{)}^{\theta_{\mathrm{cov}}}$$

(2) $$P_{ij}=\theta_{\text{pop},j}\cdot {\theta_{\mathrm{cov}}}^{\text{cat}}$$

where *P_ij_* denotes the estimate of parameter *j* in the *i*th individual, θ_Pop,*j*_ represents population value for the parameter *j*, cov_ind_ represents the individual covariate value, cov_med_ denotes the median covariate value for the population, θ_cov_ is a parameter that denotes the covariate effect, and cat is a categorical variable that takes a value of either 0 or 1 for the categorical covariates (gender, formulation, health status) analyzed. For example, the categorical variable can take a value of unity when the formulation is a tablet (cat = 0) and θ_cov_ when the formulation is a capsule (cat = 1).

The covariate analysis in NONMEM compared nested models based on likelihood ratio tests, assuming nested models. With the likelihood ratio test, the difference in the OBJFV is assumed to have a chi-square (χ^2^) distribution. A reduction in the OBJFV of ≥6.64 for a χ^2^ significance of <0.01 for 1 degree of freedom (df) using first-order conditional estimation method with interaction (FOCE-I) was considered significant. After the full model was defined, the significance of each covariate was tested individually by removal of one covariate at a time from the full model. A covariate was retained in the model if, upon removal, the OBJFV increased by more than 3.8 points (χ^2^ < 0.05 for 1 df) using FOCE-I (7).

### Model performance and validation.

Model performance for the final POP PK tafenoquine model was performed using several approaches, as listed below.

### (i) Bootstrapping.

The internal stability of the model was tested with the bootstrap approach. The analyses data were sampled with replacement at the subject level to generate 500 data sets. The final POP PK model was then bootstrapped with these 500 data sets. Median parameter estimates and 90% confidence intervals were calculated and compared with those obtained from the final model.

### (ii) VPCs.

Visual predictive checks (VPCs) (500 replications) were performed with the base and final POP PK model parameters to compare the distribution of the simulated POP PK data (median, 95% confidence interval) to the observed data. The VPC of the final model was performed using parameter estimates from the bootstrap runs using the mrgsolve package in R. This approach enabled trial simulations by including the uncertainty in parameter estimates. The VPCs were also stratified by study, doses, and other key covariates of relevance (e.g., formulation, ethnicity, health status) to evaluate the model prediction of the observed data at a more granular level.

### (iii) External data set.

Another rigorous approach to validate the final POP PK model performance was to utilize the GATHER study data, which were not used in the data set used for parameter estimation. Five hundred clinical trials were simulated using 500 vectors of bootstrap parameter estimates from the final POP PK model with a simulation data set comprising GATHER study subject demographics and the mrgsolve package in R. The median and 95% prediction intervals from these simulations were overlaid with the observed data from the GATHER study data set to assess the model performance in predicting the concordance with tafenoquine concentrations from the GATHER study.

### Relative bioavailability across formulations.

The final POP PK model was utilized to characterize any differences in relative bioavailability across formulations utilizing different approaches, as described below.

First, the relative bioavailability of the tablet and capsule formulations was estimated as a population parameter (*F*1) in the final POP PK model. The parameter point estimate and precision around the estimate were obtained by the bootstrap approach (*n* = 500). For the second approach, the tafenoquine exposures were computed via the individual *post hoc* PK parameters obtained from the final POP PK model and compared using components of traditional BE analysis across the DETECTIVE part 1 (capsule formulation), DETECTIVE part 2 (tablet formulation), and GATHER (tablet formulation) studies. For the third approach, predicted exposures obtained from the 500 bootstrap estimates were compared between these patient studies.

## Supplementary Material

Supplemental file 1
