# Supplementary material for: Population Pharmacokinetics of Tafenoquine, a Novel Antimalarial
Source: Antimicrob Agents Chemother. 2018 Oct 24;62(11):e00711-18. doi: 10.1128/AAC.00711-18 (PMC6201082; doi:10.1128/AAC.00711-18)
Supplement: Supplemental file 1 [file zac011187619s1.pdf]

Supplementary Tables

Table S1. Summary of demographics for subjects included in the validation dataset (GATHER)

**Parameter**	**Median (range)**	**Category**	**Count (%)**
Age (years)	36.0 (16.0-75.0)		
Weight (kg)	64.8 (38.0-122.8)		
Body Mass Index (kg/m^2)	24.9 (16.7-48.9)		
Gender		Female	52.0 (31.3%)
		Male	114 (68.7%)
Race		African American	2.00 (1.20%)
		Asian	41.0 (24.7%)
		American Indian/ Alaskan native	87.0 (52.4%)
		Multiple	36.0 (21.7%)

Table S2. Predicted Exposures obtained based on 500 bootstrap model runs

**Study**	**Formulation**	**Dose (mg)**	**AUC_{0-60} (ug*h/mL) Median (5th-95th percentile)**	**C_{max} (ng/mL) Median (5th-95th percentile)**
DETECTIVE Part I	Capsule	300	93.19 (56.10-150.62)	296.98 (168.16-518.41)
DETECTIVE Part II	Tablet	300	101.66 (57.25-172.80)	322.42 (167.10-592.56)
GATHER	Tablet	300	100.14 (58.05-168.47)	315.45 (170.27-574.28)

AUC_{0-60} represents the AUC from 0 to 60 days.

Table S3. Summary PK sampling scheme for different studies

Study	PK sampling times
200951 (DDI)	2, 6, 12, 24, 48, 60 and 72 hours post-dose and Days 7, 14, 21, 28, and 56
201780 (SIL)	1, 2, 6, 9, 12, 15, 20, 24, 36, 48, 60, and 72 hours post-dose and Days 7, 14, 21, 28, and 56
TAF114582 (TQT)	1, 2, 3, 4, 5, 6, 9, 12, 15, 20, 24, 36, 48 and 72 hours post dose
TAF112582 (DETECTIVE part 1)	6-12 h, 24-48 h post dose, and days 8, 15, 29 and 60[a]
TAF112582 (DETECTIVE part 2)	6-12 h, 24-48 h post dose, and days 8, 15, 29 and 60 [a]
TAF116564 (GATHER)	6-12 h, 24-48 h post dose, and days 8, 15, 29 and 60 [a]

[a]A time window of ±1, 2, 3, 7 days applies to the sampling on days 8, 15, 29, and 60 visits, respectively.

Fig. S1 Including Health Status as a covariate on V2/F

A.

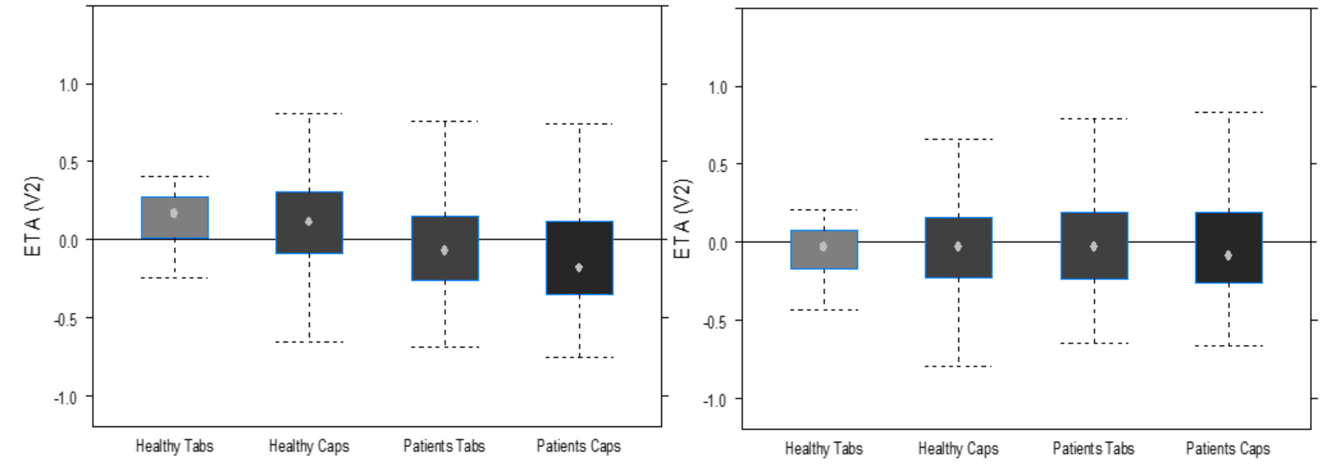

B

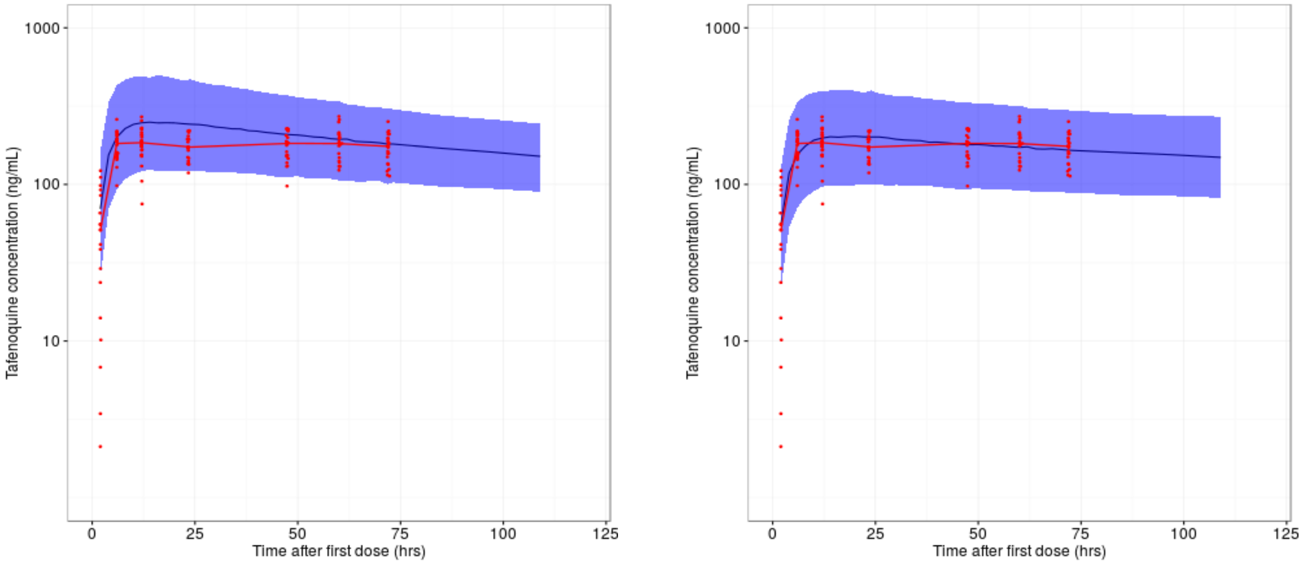

A: Improvement in the ETA(V2/F) plots before (left panel) and after (right panel) addition of health status on V2/F. (Healthy Caps- healthy volunteer study with capsule formulation, Healthy Tabs - healthy volunteer study with tablet formulation, similarly Patient Caps and Patient Tabs)

B: Improvement in the TQ exposure predictions with the DDI study at early timepoints before (left panel) and after (right panel) inclusion of health status on V2/F. The shaded region is the 95% prediction interval with solid blue line as the predicted median. The red points are observed data and the red line is the observed median.

Fig S2. Visual Predictive Checks for final model (A) across different doses of TAF112582 (DETECTIVE Part 1), (B) 201780 (SIL), (C) TAF114582 (TQT)

(A)

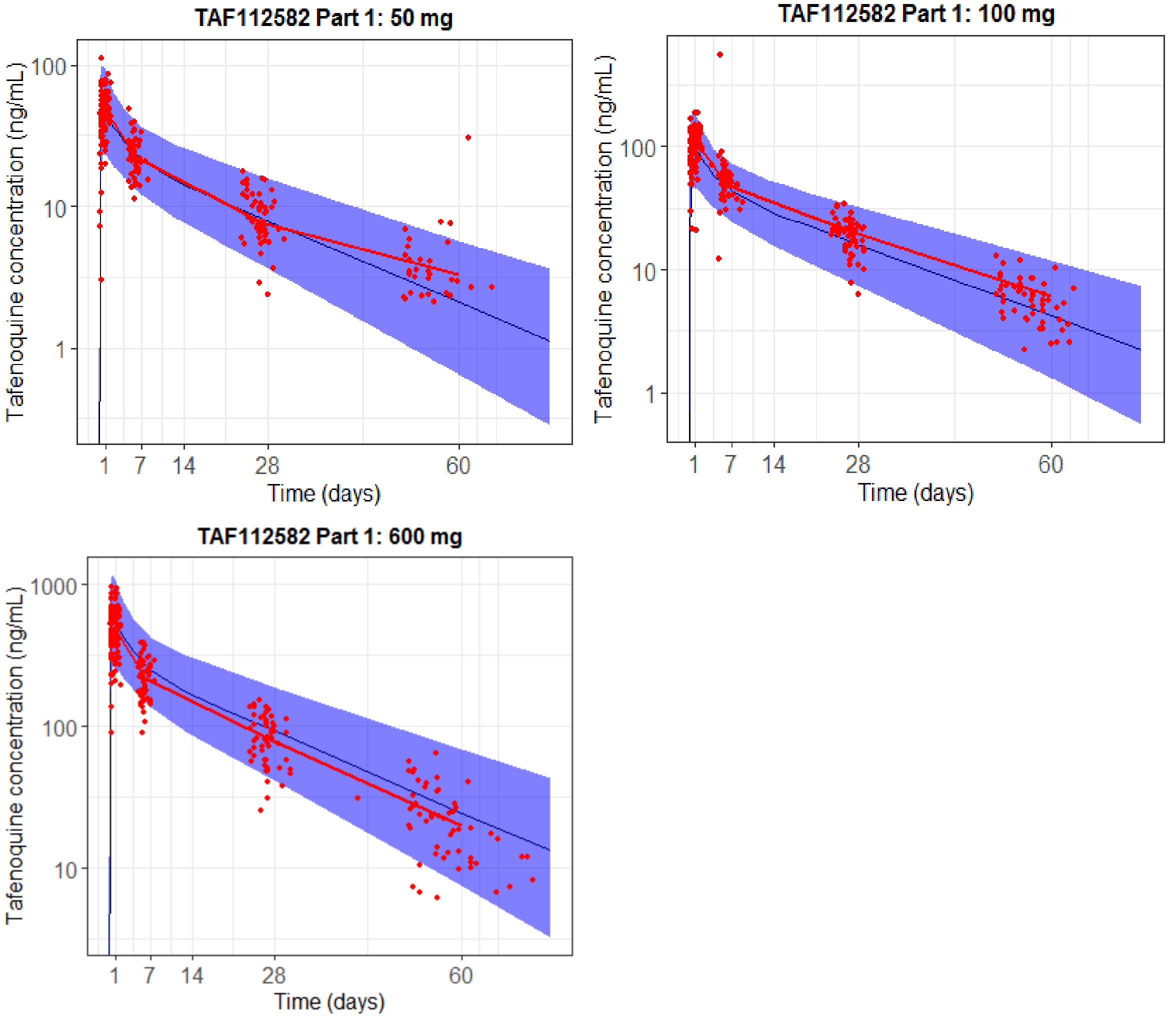

(B)

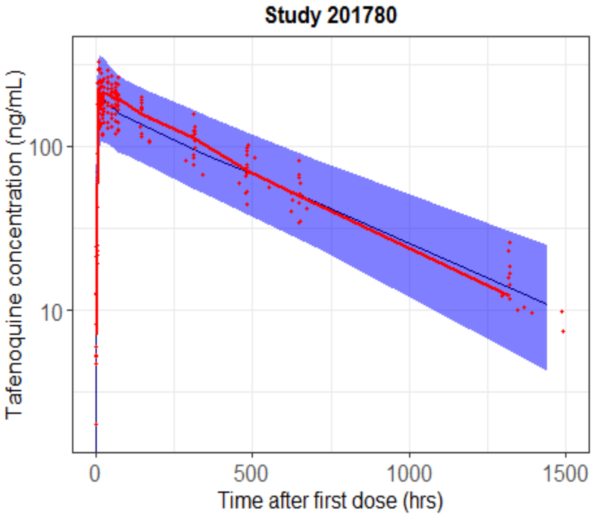

(C)

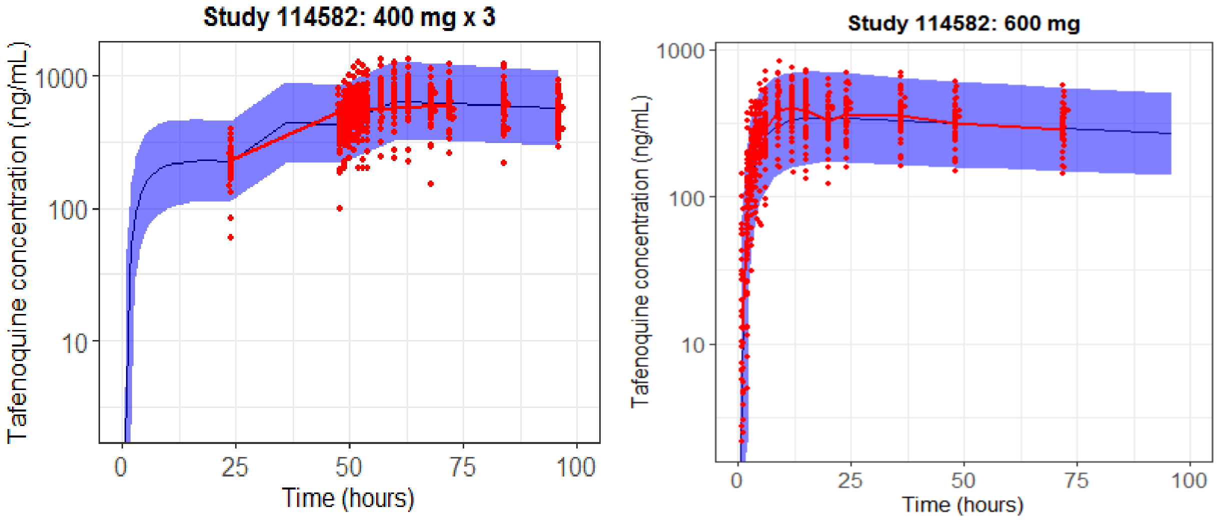

The blue bands and lines represent the 95 percent prediction intervals and median prediction, respectively. The red dots and red line represent the observed data and observed median, respectively.
